# Supplementary material for: Risk factors for sacrococcygeal pilonidal sinus: a systematic review and meta-analysis supplemented by genetic causal assessment
Source: Front Surg. 2026 Jan 7;12:1718589. doi: 10.3389/fsurg.2025.1718589 (PMC12819706; doi:10.3389/fsurg.2025.1718589)
Supplement: Supplementary file 2 [file Datasheet2.zip › Supplementary Data 2/MR_pipeline_p5e-8/forest_plots/ukb-a-224.finngen_R12_L12_PILONIDALCYST.pdf]

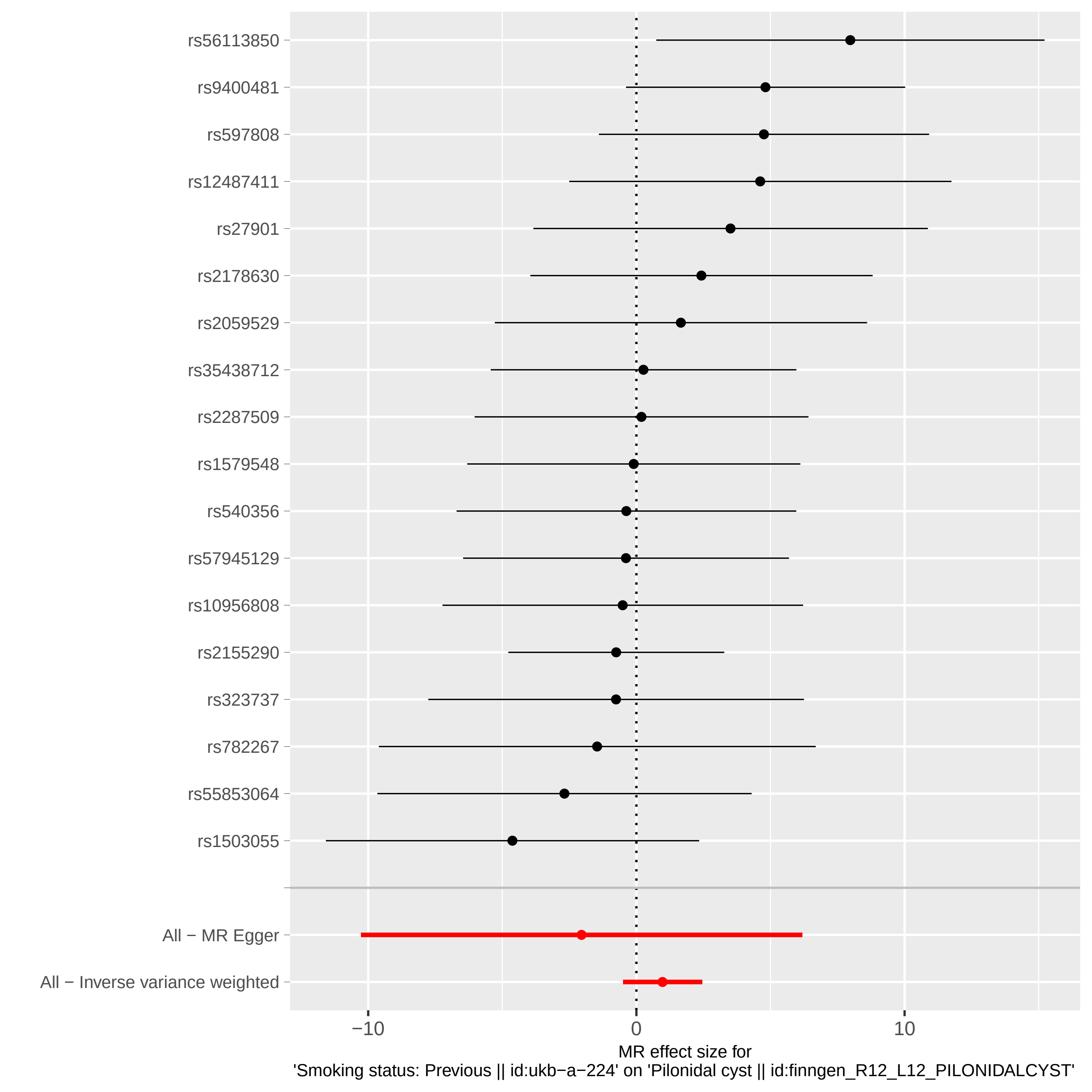

rs56113850
rs9400481
rs597808
rs12487411
rs27901
rs2178630
rs2059529
rs35438712
rs2287509
rs1579548
rs540356
rs57945129
rs10956808
rs2155290
rs323737
rs782267
rs55853064
rs1503055
All − MR Egger
All − Inverse variance weighted
−10
0
10
MR effect size for
'Smoking status: Previous || id:ukb−a−224' on 'Pilonidal cyst || id:finngen_R12_L12_PILONIDALCYST'
